# A novel lncRNA-miRNA-mRNA triple network identifies lncRNA *TWF1* as an important regulator of miRNA and gene expression in coronary artery disease

**DOI:** 10.1186/s12986-019-0366-3

**Published:** 2019-06-06

**Authors:** Liu Miao, Rui-Xing Yin, Qing-Hui Zhang, Xi-Jiang Hu, Feng Huang, Wu-Xian Chen, Xiao-Li Cao, Jin-Zhen Wu

**Affiliations:** 1 Department of Cardiology, Institute of Cardiovascular Diseases, The First Affiliated Hospital, Guangxi Medical University, 6 Shuangyong Road, Nanning, 530021 Guangxi People’s Republic of China; 2 Guangxi Key Laboratory Base of Precision Medicine in Cardio-cerebrovascular Disease Control and Prevention, 6 Shuangyong Road, Nanning, 530021 Guangxi People’s Republic of China; 3 Guangxi Clinical Research Center for Cardio-cerebrovascular Diseases, 6 Shuangyong Road, Nanning, 530021 Guangxi People’s Republic of China; 4 Department of Neurology, The First Affiliated Hospital, Guangxi Medical University, 6 Shuangyong Road, Nanning, 530021 Guangxi People’s Republic of China

**Keywords:** Coronary artery disease, LncRNA-miRNA-mRNA regulators, Triple network analyses, Function enrichment, Biomarker of diagnosis

## Abstract

**Background:**

Long non-coding RNAs (lncRNAs) are involved in numerous physiological functions. Yet, their mechanisms in coronary artery disease (CAD) are not well understood.

**Methods:**

The expression profile of genes associated to CAD was reannotated into the lncRNA-mRNA biphasic profile. The target microRNA data were used to design a global CAD triple network. Thereafter, we conducted a functional enrichment analysis and clustering using the triple network from the level of topology analyses. The expression of four non-coding RNAs (ncRNAs) was measured by qRT-PCR and the risk of CAD was calculated by nomogram. The prognostic value of three ncRNAs was evaluated using receiver operating characteristic (ROC) curve.

**Results:**

A CAD lncRNA-miRNA-mRNA network was constructed which included 15 mRNAs, 3 miRNAs, 19 edges and one lncRNA. Nomogram showed that four ncRNAs were the risk of CAD. After RT-PCR validation in four ncRNAs between CAD and non-CAD samples, only three ncRNAs had significant meaning for further analysis. ROC curve showed that *TWF1* presented an area under curve (AUC) of 0.862, the AUC of hsa -miR-142-3p was 0.856 and hsa -miR126-5p was 0.822. After the pairwise comparison, we found that *TWF1* had significant statistical significance (*P*_TWF1–142_ < 0.05 and *P*_TWF1–126_ < 0.01**)**. The results of functional enrichment analysis of interacting gene and microRNA showed that the shared lncRNA *TWF1* may be a new factor for CAD.

**Conclusions:**

This investigation on the regulatory networks of lncRNA-miRNA-mRNA in CAD suggests that a novel lncRNA, lncRNA *TWF1* is a risk factor for CAD, and expands our understanding into the mechanisms involved in the pathogenesis of CAD.

**Electronic supplementary material:**

The online version of this article (10.1186/s12986-019-0366-3) contains supplementary material, which is available to authorized users.

## Background

Coronary artery disease (CAD) remains one of the most common causes of death worldwide, killing nearly 17 million people each year [1]. In China, it was recently reported that approximately 700,000 deaths from CAD are recorded annually [2]. CAD, as a complex and multifactorial disorder, was caused by lots of environmental exposures and genetic factors, including gender difference, age, dyslipidemia, hypertension, diabetes, obesity, smoking behavior, and family history [3–6]. Early identification of CAD at high risk and adverse cardiovascular outcomes, using circulating or imaging biomarkers, may help in treatment [7]. However, currently available CAD biomarkers have limited risk prediction [8, 9].

There is increasing evidence that most non-coding RNAs (ncRNAs) have important functions in the modulation of physiological and pathological processes [10, 11]. There are two classes of ncRNA according to the number of nucleotides, including short ncRNAs (< 200 nucleotides) e.g., transcription initiation RNAs, PIWI-interacting RNAs and microRNAs and long lncRNAs (> 200 nucleotides) e.g., enhancer-like ncRNAs and transcribed ultra-conserved regions, natural antisense transcripts and long intergenic ncRNAs (lincRNAs) [12–14]. Unlike the highly conserved short ncRNA which inhibits post-transcription, lncRNAs are involved in diverse processes and are not highly conserved [15, 16].

Many studies have shown that many ncRNAs play key roles in specific physiological and pathological processes of CAD [17, 18]. Because of their stability in blood and other body fluids, ncRNAs would be served as biomarkers for cardiovascular diseases (CVDs). Therefore, we hypothesized that there are specific circulating ncRNAs that may serve as CAD biomarkers. Here, a lncRNA-miRNA-mRNA network related to CAD was constructed by mapping the differentially expressed mRNAs, miRNAs and lncRNAs into a global triple network via Gene Expression Omnibus (GEO) repository [19] and want to identify which ncRNAs would be a sensitive and specific marker of CAD.

## Materials and methods

### Reannotation of gene expression profile probe

**Fig. 1:**
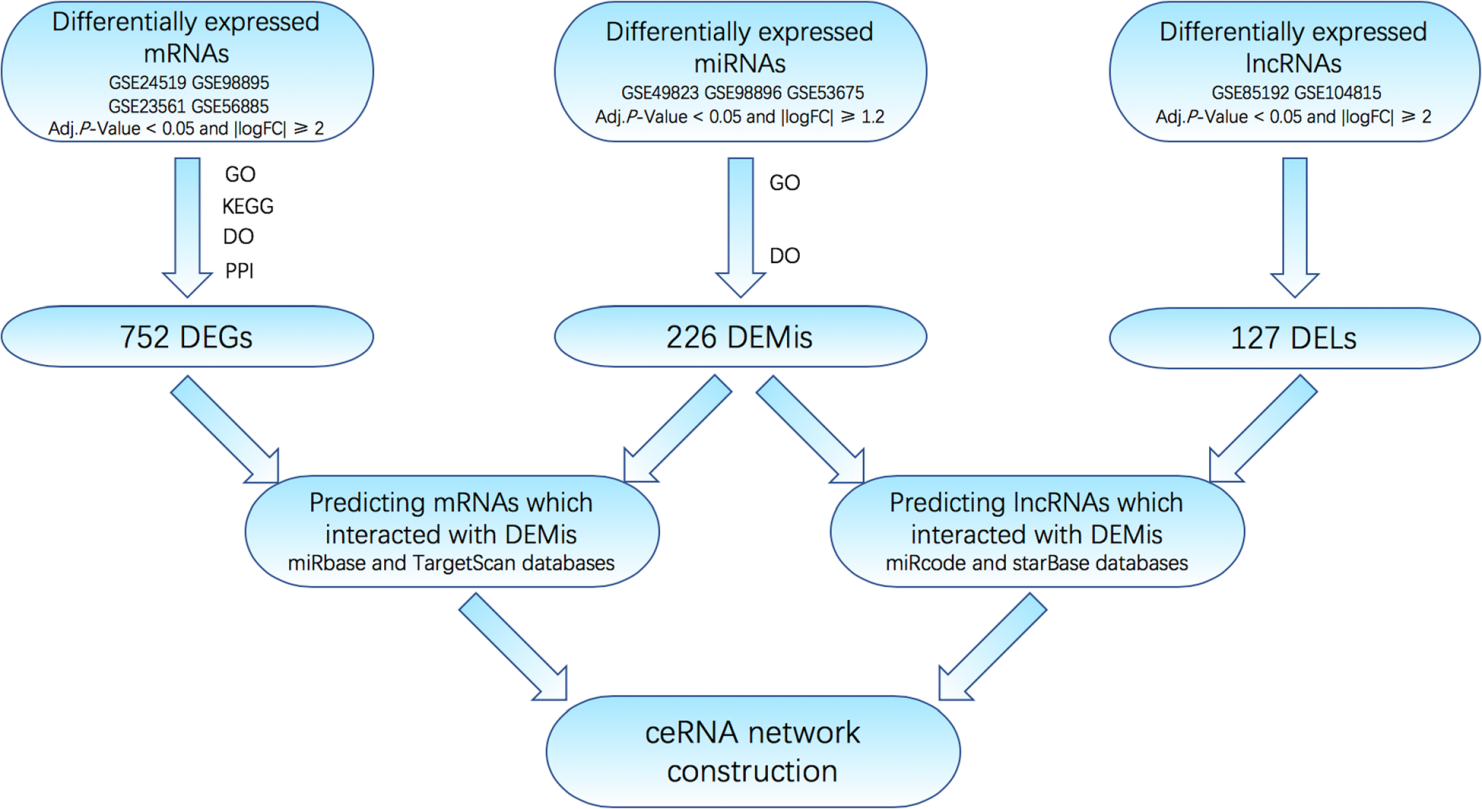
A flowchart of competing endogenous RNAs (ceRNAs) network construction. (1) DEGs and DELs with fold change ≥2.0 and adjust *P*-value < 0.05 were used, whereas DEMis was used with fold change ≥1.2 and adjust *P*-value < 0.05; (2) mRNAs-miRNAs interactions were predicted by miRbase and TargetScan databases; (3) miRNA-lncRNA interactions were predicted by miRcode and starBase databases; (4) lncRNAs which were not associated with DEMis were removed; (5) mRNAs that were not matched DEMis were removed; (6) ceRNA network was constructed

A total of 9 gene expression profile datasets were downloaded from the Gene Expression Omnibus database (https://www.ncbi.nlm.nih.gov/geo/). The 9 datasets were selected for further bioinformatic analysis, including GSE24519 [20], GSE98895 [21], GSE23561 [22] and GSE56885 for mRNAs; GSE49823, GSE98896 [21] and GSE53675 [23] for miRNAs; and GSE85192 and GSE104815 [24] for lncRNAs. All of the datasets were related to CAD. The transcript sequences of the lncRNAs and proteins were downloaded from the GENCODE database (http://www.gencodegenes.org/). Probe annotation sequence was based on previous reports [25]. Protein coding sequences and probe-matched lncRNA were obtained from Blastn tools. Ideal alignment results were screened based on the following criteria: reserve the probes that: 1) perfectly matched to more than three probes; 2) matched exclusively to a single transcript in probe-transcript pairs; 3) matched to lncRNA transcripts or protein coding transcripts [26]. Affy package in R was used to convert the CEL file to expression value matrix [27], and was quantile normalized using Robust Multi-array Average (RMA) method. Then use the Bioconductor in R to convert the probe information into a gene name [28]. The mean expression value was used for a gene with multiple probes. The specific workflow is shown in Fig. 1.

### Differentially expressed mRNAs (DEGs), miRNAs (DEMis) and lncRNAs (DELs) and functional enrichment analysis

DEGs, DEMis and DELs were selected using the limma package [29] in R software in all samples. The threshold values for DEGs and DELs were adjusted *P* < 0.05 and |log_2_fold-change| ≥ 2, while the threshold values for DEMis were adjusted *P* < 0.05 and |log_2_fold-change| ≥ 1.2. Using clusterProfiler, DOSE and Goplot package in R [30, 31], disease ontology (DO), genomes (KEGG) pathway, Kyoto Encyclopedia of Genes and the gene ontology (GO) analyses of DEGs, and GO and DO function analysis of DEMis were carried out. In all of analyses, statistical significance was represented by the adjusted *P-*value (Q-value) of < 0.05.

### Protein-protein interaction analysis (PPI) network construction and module analysis

The STRING database [32] provides information on protein prediction and experimental interactions. The database’s prediction methods come from neighborhoods, databases and text mining, co-expression experiments, co-occurrence and gene fusion. In addition, the interaction of protein pairs in the database was presented as a combined fraction. In this study, DEGs were mapped to PPIs, and a combined score > 0.9 was used as the cutoff value [33, 34], to analyze key genes in the network. The role of protein nodes in the network was described by degrees. Network modules were one of the cores of protein networks and may have specific biological implications. The major clustering modules were examined using the Cytoscape software package Molecular Complex Detection (MCODE) [35, 36] which was used to analyze the most notable clustering modules. Next, the clusterProfiler software package was used to examine the KEGG pathway for DEGs enrichment in different modules. EASE ≤0.05, count ≥2 was set as the cutoff value, and the threshold for the subsequent analysis was MCODE score > 6.

### Construction of lncRNA-miRNA-mRNA competing endogenous RNAs (ceRNAs) network

The construction of ceRNA network was derived from the correlation among mRNA, miRNA, and lncRNA. The interactions between lncRNA and miRNA were predicted by the starBase and miRcode databases [37, 38] which were used to predict the lncRNA-miRNA interactions. The target mRNAs of miRNAs were predicted using the miRbase and TargetScan [39, 40]. After GO, DO, KEGG, PPI and MCODE analysis, the DEGs which did not match DEMis should be removed. The same method was used for the lncRNA-miRNA interactions. The lncRNA-miRNA-mRNA ceRNA network was constructed and visualized using the Cytoscape.

### Study population

A total of 857 subjects were recruited from outpatient for a complaint of chest in the First Affiliated Hospital, Guangxi Medical University from Jan. 1, 2015 to Dec. 31, 2017. Since angiographic examination was performed for CAD and or other suspected diseases, all participants were examined using coronary angiography by two experts. CAD was defined as significant coronary stenosis (≥ 50%) in at least one of the three main coronary arteries or their major branches (branch diameter ≥ 2 mm). If the coronary stenosis does not meet the above criteria, the subject should be defined as normal coronary artery. To analyze the influence of the expression level of hub gene on the prognosis of the disease, the individuals were divided into CAD and control groups after coronary angiography. Participants who had a history of CAD, type I diabetes mellitus and congenital heart disease were excluded [41]. Medical history and general information were obtained using a standard questionnaire. Thus, the control group was judged to include the subject who with normal coronary artery and the without any excluded disease. The investigation conformed to the rules of the Helsinki Declaration of 1975 (http://www.wma.net/en/30publications/10policies/b3/), and the new edition of 2008. The research design was approved by the Ethics Committee. The First Affiliated Hospital of Guangxi Medical University (No: Lunshen-2011-KY-Guoji-001; March 7, 2011). All procedures are conducted in conformity to ethical standards. All participants singed an informed consent upon receipt of a complete explanation of the study [42]. In the initial evaluation, all clinical data were collected based on the medical records. Clinical data collection, biochemical measurements and diagnostic criteria were performed according to previous studies [43–45].

### RNA isolation and RT- quantitative PCR (qPCR)

EDTA coated tubes were used to collect fasting blood (5 mL) in the early morning. The blood was centrifuged at 3000 g for 15 min. To remove cell debris, the sample was further centrifuged at 1200 g for 15 min at 4 °C and then kept at − 80 °C. the miRNeasy serum/plasma kit (TIANGEN: Cat. No. DP503, China) was used to isolate total RNA containing miRNA. Briefly, a denaturing buffer was added to 200 μL of plasma sample according to the manufacturer’s instructions. After a 5-min incubation at room temperature, 25 fmol of synthetic cel-miR-39 (TIANGEN; catalog number: CD200–01, China) were added. Thereafter, RNA was isolated using the procedure provided by the manufacturer. RNase-free water (330 μL) was used to elute total RNA. A spectrophotometer (NanoPhotometer P300, Implen) was used to determine the level of hemolysis in plasma samples [46]. cDNA was obtained through a reverse transcription of RNA by a reverse transcriptase kit (TIANGEN; catalog number: KR211, China). The reaction mixture included 10 μL of miRNA RT reaction buffer, 2 μL of enzyme mixture, RNase-free water (up to 20 μL) and 2 μg of total RNA. The mixture was incubated at 42 °C for 60 min, at 95 °C for 3 min, and then at 4 °C. A no RT negative control was used in all tests to ensure that there was no contamination between genomic DNA and PCR product. Quantification of three plasma miRNAs was performed by real-time PCR based on SYBR Green using the miScript SYBR Green PCR Kit (TIANGEN; Cat. No. FP411, China). The reaction contained RNase-free water (up to 20 μL), 3.0 μL cDNA, 0.4 μL PCR reverse primer, 0.2 μL PCR Forward Primer and 2 × miRcute Plus miRNA Premix 10 μL. The reaction was incubated at 95 °C for 15 min, at 94 °C for 25 s, at 60 °C for 30 s, and at 72 °C for 34 s. All reactions were performed in duplicate. Ct values were averaged for each sample after two PCRs experiments. The internal control was for the target RNAs was the cel-miR-39 [47]. The 2 ^- [Ct (miRNA)- Ct (cel-miR-39)]^ formula was used to calculate the relative expression of each miRNA after normalization to cel-miR-39. The lncRNA expression levels were quantified in triplicate. The plasma lncRNAs levels were determined by the ∆*C*_T_ method because there is, as yet, no consensus about stable and suitable internal controls for lncRNA in plasma samples. The change in gene expression was calculated using the equation. 2 ^−∆*C*T^ [48]. The relative expression level of lncRNA in monocytes was normalized to the internal control ACTB expression and calculated by the comparative *C*_T_ (∆∆*C*_T_) method. Melting curve analysis was used to determine the specific and lack of primer dimers for amplification. The primers used in qPCR of the lncRNAs and miRNAs are listed in Additional file 1 Table S1.

### Statistical analysis

The statistical software package SPSS 21.0 (SPSS Inc. Chicago, IL, USA) and Prism 5 (GraphPad Software) were used for all statistical analyses. Chi-square analysis was applied to assess differences in ratios among groups. Continuous data are presented as means ± SD [49]. For those that are usually distributed, the median and quartile ranges of triglyceride (TG) are usually not distributed. Comparison of continuous data sets using Mann-Whitney nonparametric tests and Kruskal-Wallis [50]. R software (version 3.5.0) was used to measure the heart-map of correlation models and bioinformatic analysis. The CAD risk score was calculated for each patient as a linear combination of selected predictors that were weighted by their respective coefficients. The ‘rms’ package was used for CAD prediction nomogram. The predictive accuracy of the risk model was assessed by discrimination measured by C-statistic and calibration evaluated by Hosmer-Lemeshow *χ*^*2*^ statistic. To compare the plasma miRNAs and lncRNAs between the control and case groups, receiver operating characteristic (ROC) curve analysis was conducted. The diagnostic value of the miRNAs and lncRNAs was evaluated by the area under curve (AUC). All tests were two-sided, and statistical significance was considered for *P* < 0.05.

## Results

### Data preprocessing and identified differentially expressed items

**Fig. 2:**
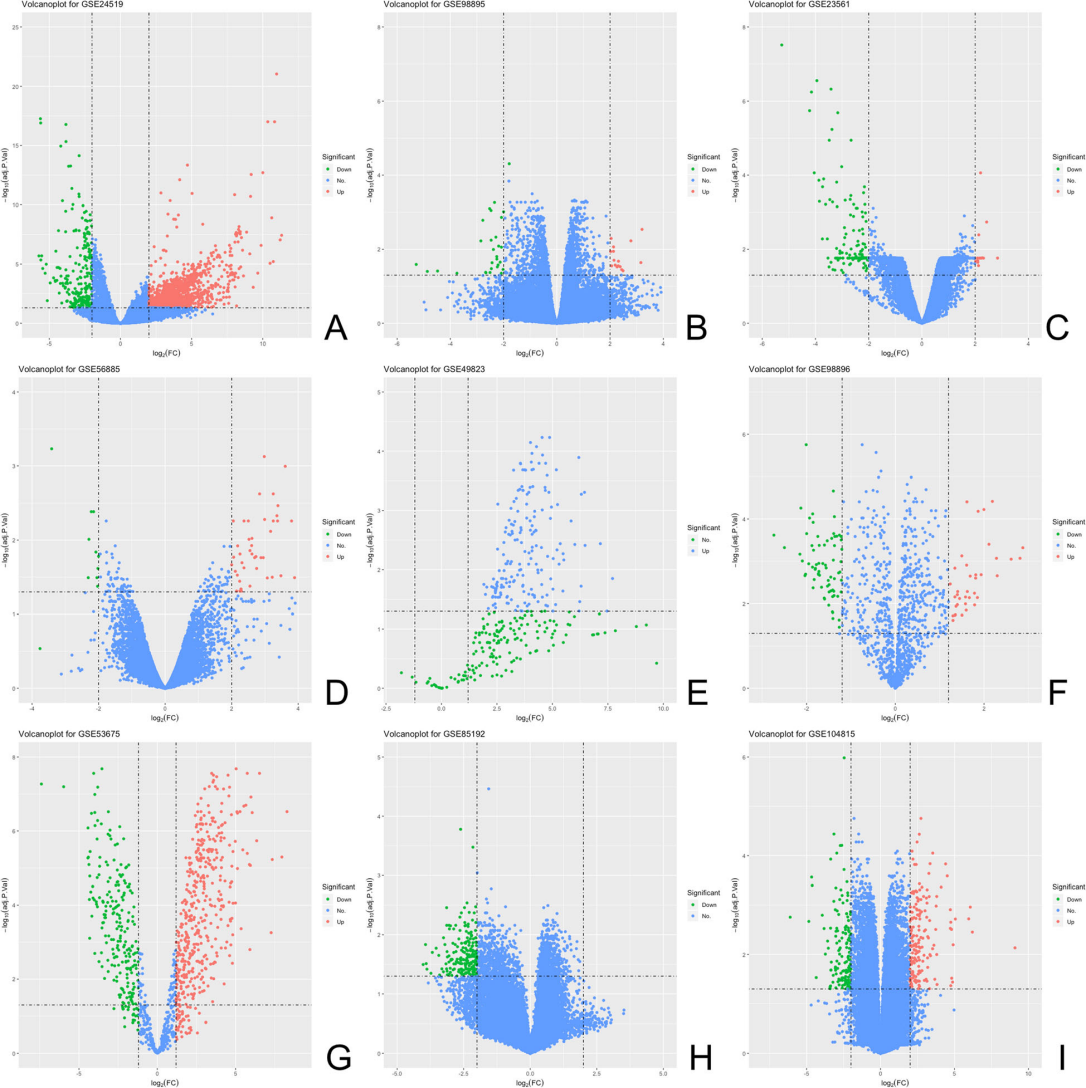
Identified DEGs, DEMis and DELs in volcanoplot. **a**-**d** DEGs; (**e**-**g**): DEMis; (**h**-**i**): DELs. The two vertical lines are the 2-fold change boundaries and the horizontal line is the statistical significance boundary (Adj-*P* < 0.05). Items with statistical significance and up-regulated are marked with red dots, and down-regulated are marked with green dots

As shown the volcano plot in Fig. 2, After quality control and get rid of lots wrong expression, we identified a total of 752 DEGs including 627 up-regulated and 125 down-regulated in CAD samples compared with control samples with adjust-*P* value (*P*_a_) < 0.05 and |log _2_ (fold change)| ≥ 2; 226 DEMis including 194 up-regulated and 32 down-regulated with *P*_a_ < 0.05 and |log _2_ (fold change)| ≥ 1.2; 127 DELs including 49 up-regulated and 78 down-regulated with *P*_a_ < 0.05 and |log _2_ (fold change)| ≥ 2 for further analysis. The detailed items are list in Additional file 1 Tables S1 to S4.

### Functional annotation, PPI network construction and identified hub items

**Fig. 3:**
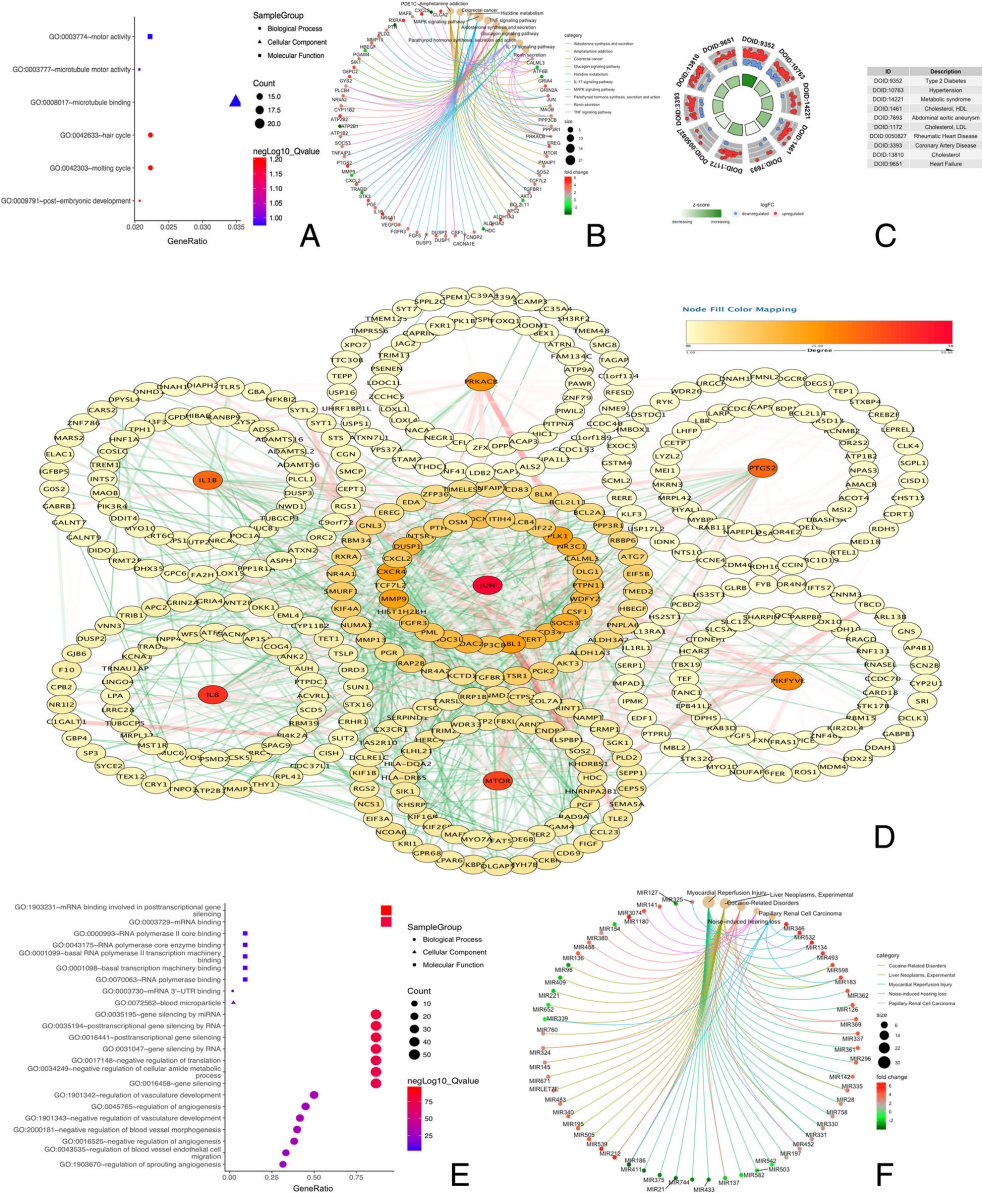
Functional annotation, PPI network construction and identified hub items. **a** GO analysis for DEGs; (**b**): KEGG analysis for DEGs; (**c**): DO analysis for DEGs. The inner ring is a bar plot where the height of the bar indicates the significance of the term and color corresponds to the z-score. The outer ring displays scatter plots of the expression levels (logFC) for the genes in each term. **d** PPI network of the selected DEGs. Edge stands for the interaction between two genes. A degree was used for describing the importance of protein nodes in the network, red shows a high degree and white presents a low degree. The significant modules identified from the PPI network using the molecular complex detection method with a score of > 6.0. **e** GO analysis for DEMis; (**f**): KEGG analysis for DEMis

To elucidate the role of DEGs, 752 genes were respectively subjected to KEGG pathway enrichment, DO functional and GO analyses by R clusterProfiler package (Fig. 3 a to c). When used the STRING database to analyze, 307 nodes and 1040 protein pairs with a combined weight score > 0.25 were screened in DEGs (Fig. 3d). When they were analyzed in sub-module, only four modules with score > 6 were detected by MCODE (Table 1). The same method was performed to analyze the miRNAs. After GO and DO functional analysis (Fig. 3 e to f), 30 hub miRNAs were identified for further analysis (Table 2).

**Table 1:** Identification of the hub genes by MCODE analysis

Gene	ID	Degree	Cluster	MCODE-score
KIF16B	55,614	6	1	8
KIF1B	23,095	7	1	8
KIF22	3835	12	1	8
KIF26B	55,083	6	1	8
KIF4A	24,137	12	1	8
RINT1	60,561	7	1	8
TMED2	10,959	9	1	8
ATG7	10,533	11	2	9
C9orf72	203,228	9	2	7.69147629
CD34	947	18	2	8.53333333
CDH10	1008	8	2	8.63333333
CHST15	51,363	8	2	9
CSF1	1435	15	2	9
CX3CR1	1524	7	2	9
CXCL2	2920	14	2	7.78571429
CXCR4	7852	24	2	7.83333333
FBXL4	26,235	7	2	9
HERC4	26,091	7	2	9
HNF1A	6927	8	2	8.6777777
JUN	3725	50	2	7.79047619
KCTD7	154,881	8	2	9
KLHL21	9903	7	2	9
LPA	4018	10	2	9
MBL2	4153	8	2	9
MMP13	4322	9	2	7.78571429
MMP9	4318	23	2	8.53333333
PTGS2	5743	33	2	8.78333333
RBBP6	5930	10	2	9
SMURF1	57,154	13	2	9
SOCS3	9021	20	2	9
SYT1	6857	9	2	9
TCF7L2	6934	7	2	8.78571429
TERT	7015	18	2	8
WDFY2	115,825	17	2	8.80555556
CTPS1	1503	8	3	6
GNL3	26,354	11	3	7
KRI1	65,095	5	3	7
NMD3	51,068	8	3	7
NOC3L	64,318	17	3	7
RBM34	23,029	9	3	6
RRP1B	23,076	8	3	7
TSR1	55,720	13	3	7
CD69	969	6	4	7
CD83	9308	9	4	6.76190476
DUSP1	1843	19	4	6.46428571
HBEGF	1839	8	4	7
IL1B	3553	33	4	7.47435897
IL8	3576	42	4	6.78333333
MTOR	2475	39	4	7.51515152
NR3C1	2908	19	4	7.57142857
OSM	5008	12	4	7.5
PGF	5228	6	4	7
PGR	5241	9	4	6.76190476

**Table 2:** Identification of the hub miRNAs

SYMBOL	miRNA-ID	Entrez-ID	log FC	Adj.P. Val
MIR126	hsa-miR-126	406,913	4.16884475	0.00230477
MIR134	hsa-miR-134	406,924	5.14494673	0.03891886
MIR142	hsa-miR-142-3p	406,934	3.28286253	0.01526726
MIR142	hsa-miR-142-5p	406,934	2.8892	5.34E-06
MIR183	hsa-miR-183	406,959	4.54685783	0.02909005
MIR186	hsa-miR-186	406,962	−3.8455	1.65E-06
MIR197	hsa-miR-197	406,974	1.90947233	0.0177346
MIR21	hsa-miR-21	406,991	−3.1893	5.22E-05
MIR28	hsa-miR-28-3p	407,020	2.50737283	0.01899183
MIR28	hsa-miR-28-5p	407,020	−1.6632748	0.00411774
MIR296	hsa-miR-296-5p	407,022	3.76085274	0.00492719
MIR330	hsa-miR-330-5p	442,902	−2.1874	7.22E-05
MIR330	hsa-miR-330-3p	442,902	2.26316142	0.01938733
MIR331	hsa-miR-331-3p	442,903	−1.5113046	0.00284768
MIR331	hsa-miR-331-5p	442,903	2.1404	0.000238
MIR335	hsa-miR-335	442,904	3.23958656	0.00176059
MIR346	hsa-miR-346	442,911	5.47421197	0.02296153
MIR361	hsa-miR-361-3p	494,323	−3.4796	7.77E-05
MIR361	hsa-miR-361-5p	494,323	4.00837885	0.00215076
MIR362	hsa-miR-362-5p	574,030	4.18753105	0.0273828
MIR369	hsa-miR-369-3p	442,914	4.1022	0.000105
MIR375	hsa-miR-375	494,324	−3.2647	6.89E-06
MIR433	hsa-miR-433	574,034	−3.0274	1.35E-06
MIR452	hsa-miR-452	574,412	2.0908	0.00204
MIR503	hsa-miR-503	574,506	−1.5748497	0.00123618
MIR532	hsa-miR-532-5p	693,124	5.17711087	0.00020688
MIR542	hsa-miR-542-3p	664,617	−1.3711984	0.00042652
MIR582	hsa-miR-582-5p	693,167	−1.7333902	0.00196603
MIR598	hsa-miR-598	693,183	4.69393814	0.00016042
MIR744	hsa-miR-744	100,126,313	−3.1435	2.34E-05

### Construction of the lncRNA-miRNA-mRNA regulatory network

**Fig. 4:**
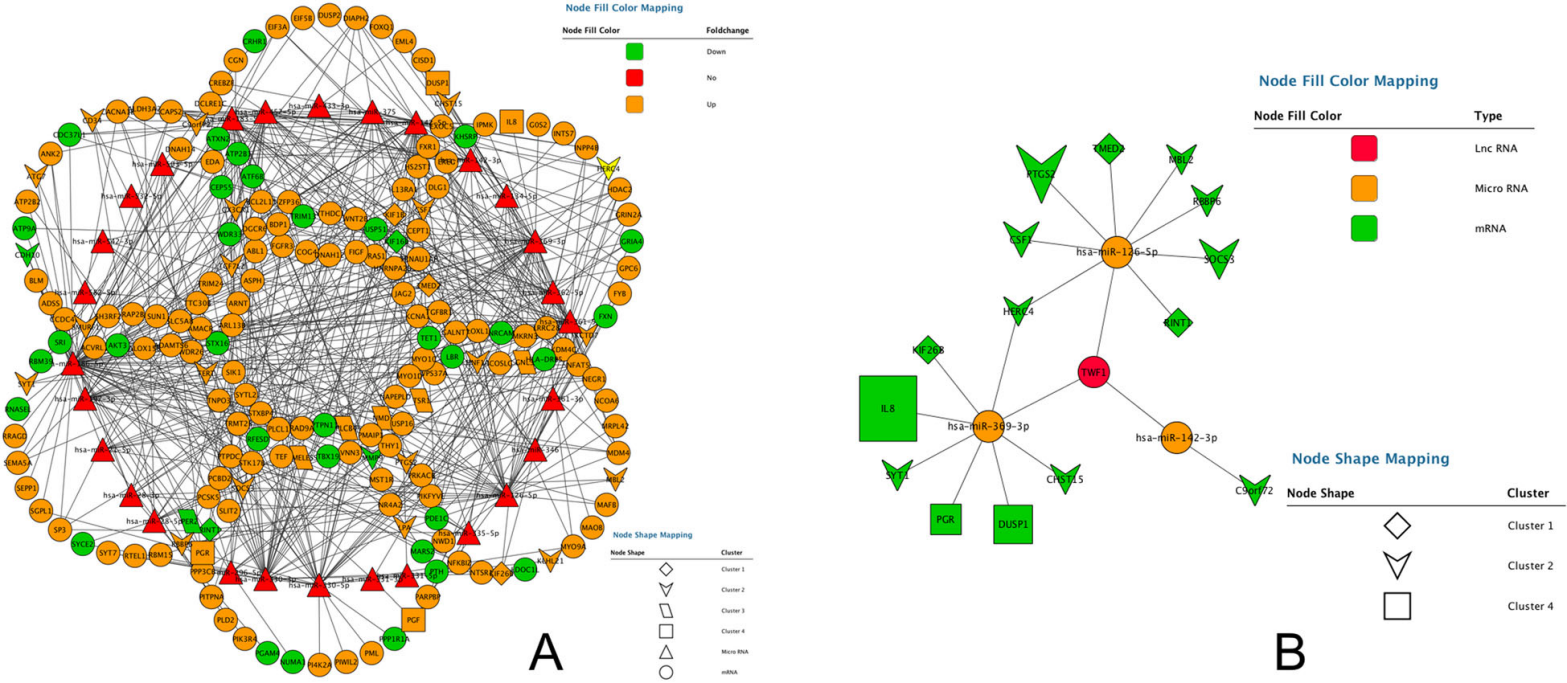
Construction of the lncRNA-miRNA-mRNA regulatory network. **a** Edge stands for the interaction between two items. Orange shows the up-regulated genes and green presents the down-regulated genes. The significant four modules identified from the PPI network shown with different shapes using the molecular complex detection method with a score of > 6.0. **b** Edge stands for the interaction between two items. Red shows lncRNAs, orange presents miRNAs and green defines as mRNAs. Different shapes show the different clusters analyzing by the molecular complex detection method

First of all, considering the interaction between mRNAs and miRNAs, the miRbase and TargetScan databases were searched for miRNA-mRNA interactions. If the predicted genes in the database were not DEGs, they would be removed. At last, we identified 520 miRNA-mRNA interaction pairs (Fig. 4a). We applied the same approach to design the lncRNA-miRNA regulatory network by starBase V2.0 and miRcode and we obtained 8 lncRNA-miRNA interactions pairs. If the predicted miRNAs in the database were not matched DELs, they would also be removed. Finally, a lncRNA-miRNA-mRNA network was formed by merging the two sets of data. LncRNAs, mRNAs and miRNAs were the nodes in the network. The edges in the network represented interactions between these RNAs [51] (Fig. 4b). In total, we found that a lncRNA (*TWF1*) and three miRNAs (hsa-miR-369-3p, hsa-miR-142-3p and hsa-miR-126-5p) were hub items in this triple regulatory network.

### Demographic characteristics, expression levels of ncRNAs and the relationship between ncRNAs and parameters related to CAD

**Fig. 5:**
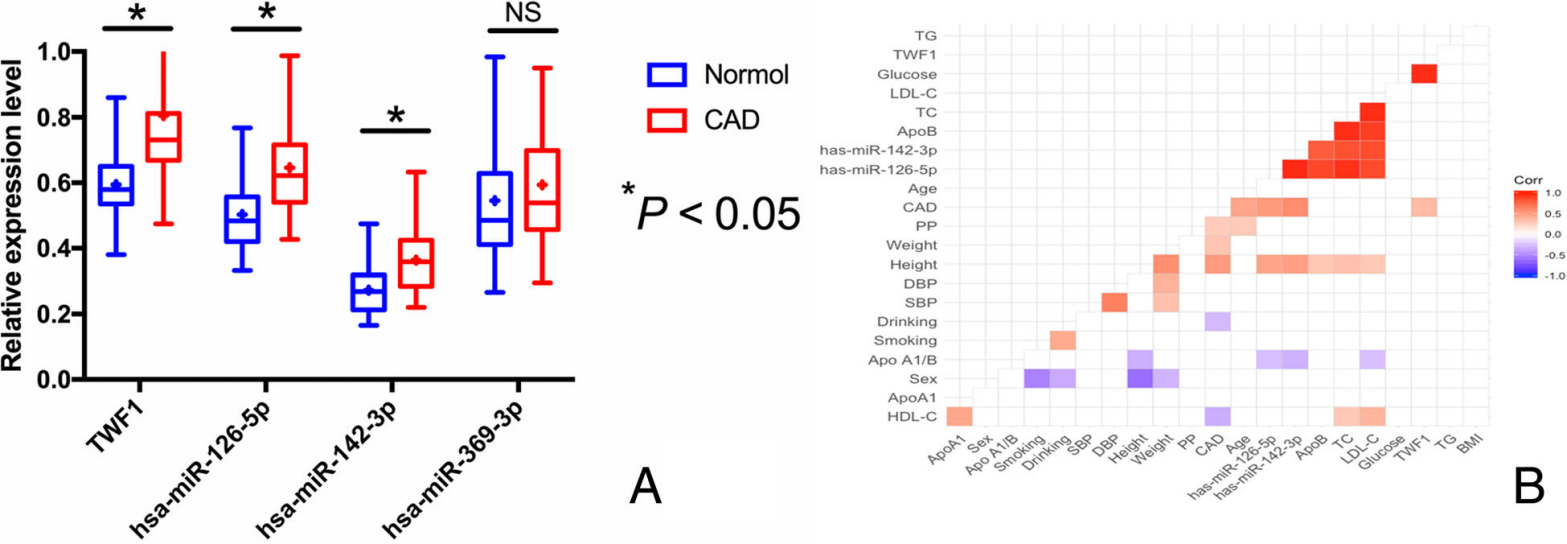
Confirmed expression levels of ncRNAs and the correlation between ncRNAs and parameters related to CAD. **a** The relative expression of circulating ncRNAs between healthy controls and CAD samples. **b** The correlation between ncRNAs and parameters related to CAD. Positive correlation is marked with red, and negative correlation are marked with blue. The colorful lattice with significant difference and leaves blank on no significant coefficient. *BMI*, Body mass index; *SBP*, Systolic blood pressure; *DBP*, Diastolic blood pressure; *PP*, Pulse pressure; *TC*, Total cholesterol; *TG*, Triglyceride; *HDL-C*, high-density lipoprotein cholesterol; *LDL-C*, low-density lipoprotein cholesterol; *Apo*, Apolipoprotein

The demographic and biochemical parameters of the participants in the two groups are presented in Table 3. The levels of weight, low-density lipoprotein cholesterol (LDL-C), total cholesterol (TC), waist circumference (WC), body mass index (BMI), blood glucose, and the percentage of cigarette smoking and suffered from diabetes were higher in CAD than in control groups. Comparison among age, gender ratio, and the percentage of alcohol drinking revealed no significant difference among groups. As compared with healthy controls, CAD patients had higher circulating *TWF1*, hsa-miR-126-5p and hsa-miR-142-3p levels (*P* < 0.05; Fig. 5a). As shown in Fig. 5b, circulating *TWF1*, hsa-miR-126-5p and hsa-miR-142-3p levels were positively correlated with CAD (*P* < 0.05 for all).

**Table 3:** Comparison of demographic, lifestyle characteristics and serum lipid levels between the normal and CAD groups*. HDL-C*, high-density lipoprotein cholesterol; *LDL-C*, low-density lipoprotein cholesterol; *Apo*, Apolipoprotein. ^1^Mean ± SD determined by *t*-test.^2^Because of not normally distributed, the value of triglyceride was presented as median (interquartile range), the difference between the two groups was determined by the Wilcoxon-Mann-Whitney test

Parameter	Control	CAD	*test-statistic*	*P*
Number	424	433		
Male/female	128/296	141/292	0.672	0.412
Age (years)^1^	55.31 ± 10.52	55.87 ± 11.13	0.987	0.378
Height (cm)	156.23 ± 6.91	155.53 ± 7.03	1.596	0.212
Weight (kg)	52.86 ± 7.84	60.74 ± 10.84	21.439	1.72E-005
Body mass index (kg/m^2^)	29.59 ± 3.23	32.31 ± 6.54	32.224	2.48E-008
Waist circumference (cm)	73.43 ± 6.61	87.45 ± 9.87	23.122	3.34E-005
Smoking status [*n* (%)]				
Non-smoker	313(74.0)	288(65.2)		
Smoker	110(26.0)	154(34.8)	7.690	0.005
Alcohol consumption [*n* (%)]				
Non-drinker	322(76.1)	330(74.5)		
Drinker	101(23.9)	113(25.5)	0.309	0.578
Systolic blood pressure (mmHg)	128.24 ± 18.18	129.47 ± 22.16	0.403	0.421
Diastolic blood pressure (mmHg)	81.54 ± 10.16	82.49 ± 13.15	0.807	0.393
Pulse pressure (mmHg)	49.64 ± 14.13	50.42 ± 14.59	1.492	0.233
Glucose (mmol/L)	5.92 ± 1.86	7.45 ± 2.25	18.817	5.93E-005
Total cholesterol (mmol/L)	4.91 ± 1.13	5.24 ± 1.07	8.121	0.019
Triglyceride (mmol/L)^2^	1.49(0.51)	1.53(1.22)	2.137	0.187
HDL-C (mmol/L)	1.52 ± 0.44	1.06 ± 0.26	8.673	0.013
LDL-C (mmol/L)	2.84 ± 0.84	2.88 ± 0.79	9.497	0.007
ApoA1 (g/L)	1.23 ± 0.25	1.17 ± 0.27	0.384	0.518
ApoB (g/L)	0.83 ± 0.19	0.89 ± 0.32	1.542	0.193
ApoA1/ApoB	1.67 ± 0.50	1.66 ± 0.57	0.095	0.758
Diabetes [*n* (%)]	47(11.3)	64(16.7)	5.192	0.023
Hypertension [*n* (%)]	197(45.4)	213(48.4)	0.795	0.373

### Nomogram risk model development to estimate individual CAD probability

**Fig. 6:**
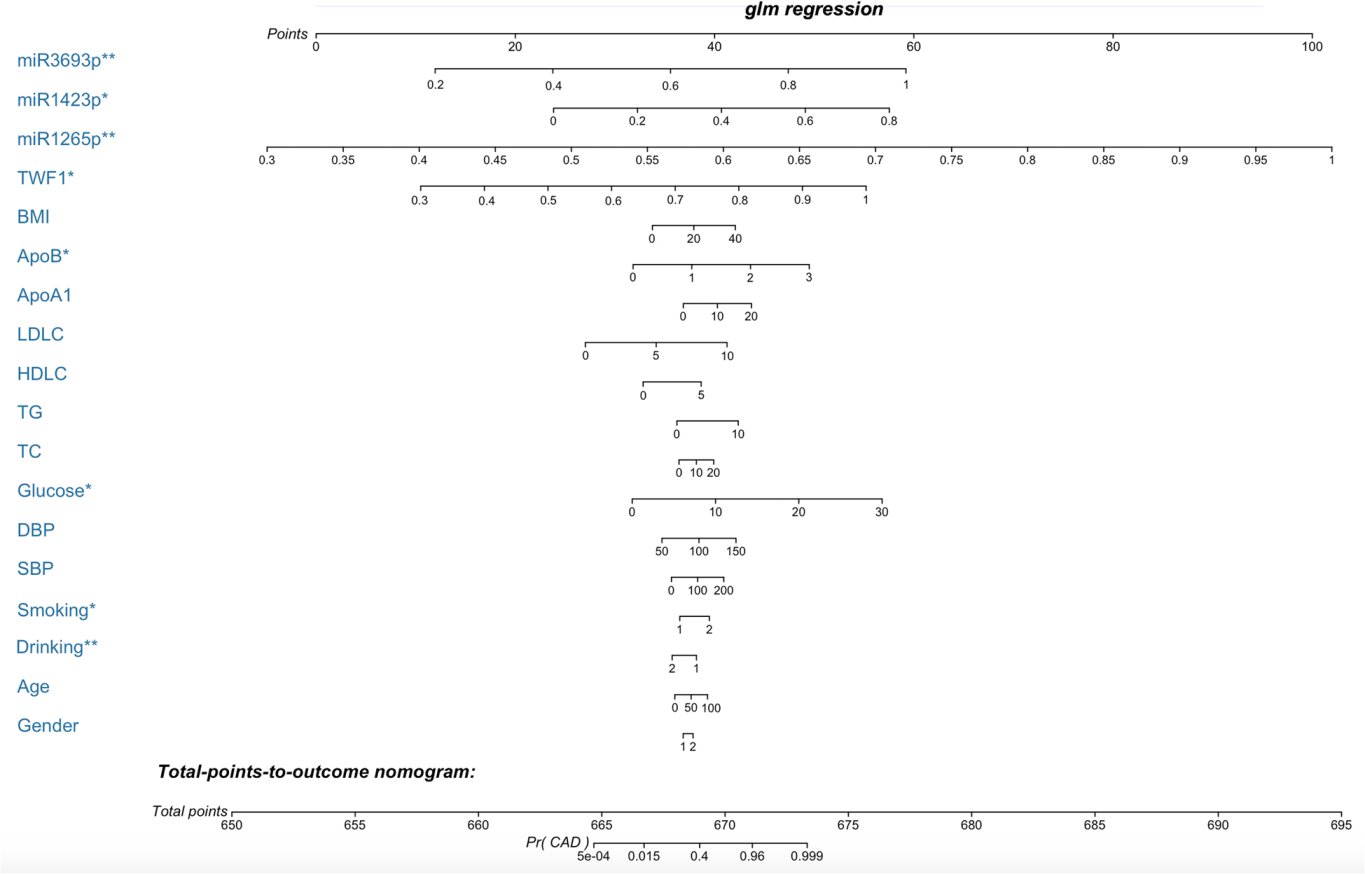
Nomogram to estimate individual CAD probability. The nomogram was constructed based on logistic regression model for outcome of definite CAD. Each predictor variable characteristic has a corresponding point value based on its position on the top point scale and contribution to the model. The probability of CAD for each subject is calculated by adding of the points for each variable to obtain a total point value which corresponds to a probability of CAD from the scale presented on the bottom line. *P < 0.05, **P < 0.01

We selected gender, age, smoking, drinking, BMI, systolic blood pressure (SBP), diastolic blood pressure (DBP), serum glucose, TC, TG, high-density lipoprotein cholesterol (HDL-C); LDL-C, apolipoprotein (Apo)A1, ApoB, the relative expression of lncRNA *TWF1*, miR-126-5p, miR-142-3p and miR-369-3p were the best subset of risk factors to develop the CAD risk score and risk model (nomogram) (Fig. 6). Where, male was labeled as 1 and female was labeled as 2; for smoking and drinking, yes was labeled as 2, no was labeled 1. The nomogram had excellent discriminative power with a C-statistic and was well calibrated with Hosmer-Lemeshow *χ*^*2*^ statistic. The predicted probabilities of developing CAD ranging from 0.000054 to 99.9%. After calculation, smoking, drinking serum glucose, ApoB, the relative expression of lncRNA *TWF1*, miR-126-5p, miR-142-3p and miR-369-3p was significantly related to the risk of CAD, with statistical significance.

### Plasma ncRNAs level sensitive for CAD

**Fig. 7:**
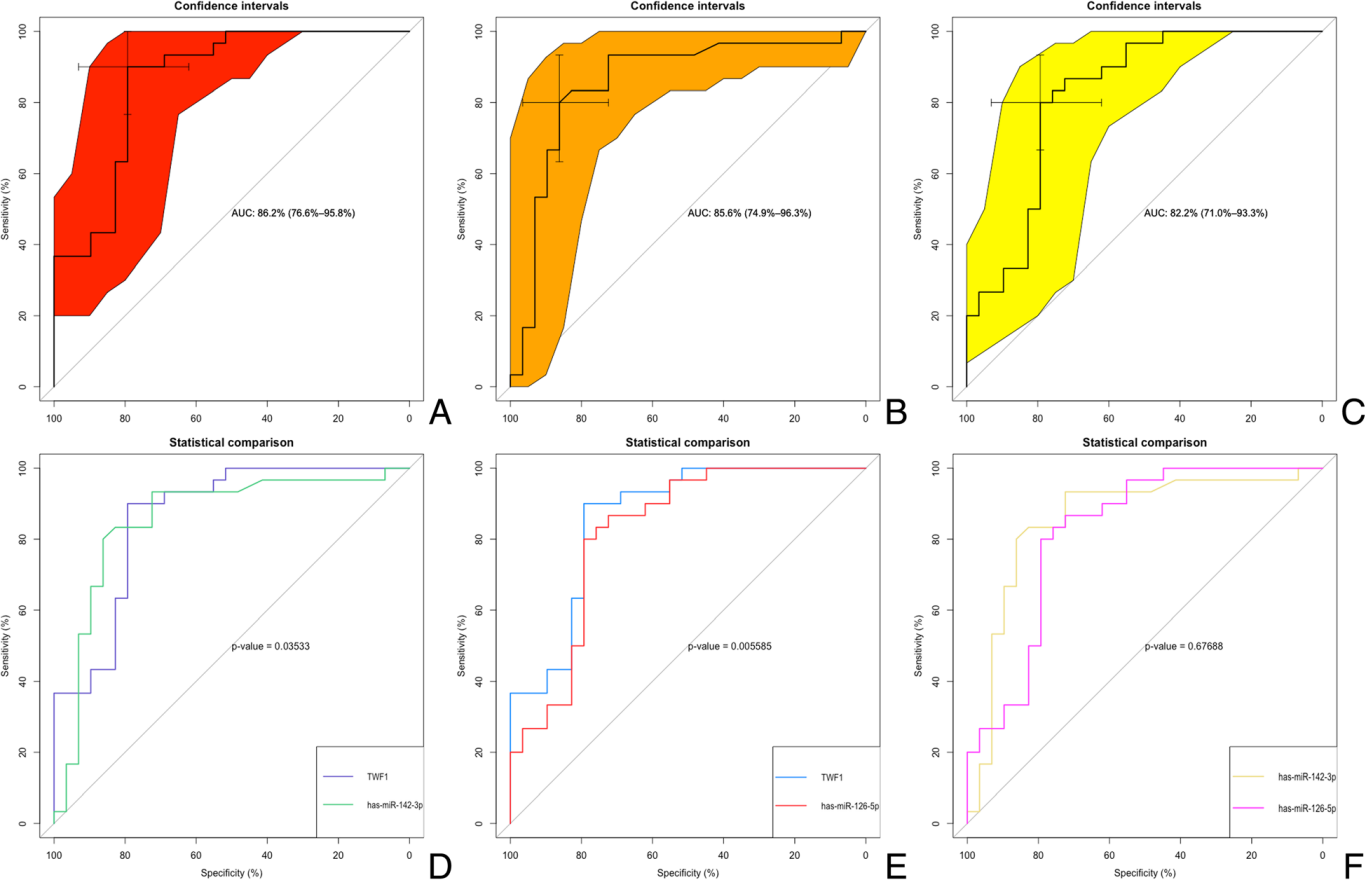
ROC curve analyses of these 3 ncRNAs for the diagnosis of CAD**. a**-**c** ROC curve analysis of TWF1, hsa-142-3p and hsa-126-5p. **d**-**f** The pairwise *P*-value comparison

As the above-mentioned observations were done, we further assessed these 3 ncRNAs as a marker for CAD. ROC curve analysis showed that *TWF1* presented an 95% confidence interval (CI) 0.766–0.958 and AUC of 0.862, the AUC of hsa-miR-142-3p was 0.856 and 95%CI 0.749–0.963 and hsa-miR126-5p was 0.822, 95%CI 0.710–0.933 (Fig. 7a). After the pairwise comparison, we found that *TWF1* had significant statistical significance (*P*_TWF1–142_ < 0.05; *P*_TWF1–126_ < 0.01, Fig. 7b).

## Discussion

Novel therapeutic strategies based on mechanisms are required to protect the heart from acute and chronic wound following CVDs. However, the mortality rate remains high. The obvious reason is that CAD is not easy to be found in its earlier stage by regular examinations, such as electrocardiography, cardiac ultrasound. Therefore, it is necessary to detect CAD in its early stage. Emerging evidence has indicated that ncRNAs, such as numerous lncRNAs and miRNAs, modulate many pathophysiological processes in development of CVDs. Indeed, ncRNA in terms of tRNA was identified and its structure was published in the early 1960s [52], accounting for around 97% of the entire genome, while the rest (∼3%) was protein coding genes, since then the research of transcriptomics has expanded and indicated that ncRNAs participated in a wide variety of cellular mechanisms and regulation processes in CVDs [53].

LncRNA belongs to a new class of non-coding RNAs, which are usually defined as transcripts longer than 200 nucleotides and have no protein coding ability [12, 14]. LncRNAs have been implicated in basic biological processes e.g., post-transcriptional gene regulation, gene transcription, chromatin modification, gene regulation and RNA processing [54]. Several lncRNAs are involved in several human diseases e.g., CVD and cancers [55, 56]. For instance, increased lncRNA *MEG3* levels may play a critical role in regulating proliferation of endothelial cells and expression of type I collagen, type V collagen resulted in atherosclerosis [57].

MiRNAs play crucial roles in the pathogenesis of CVDs; these miRNAs have also been reported as biomarkers for the prognosis and diagnosis of CAD [58]. Although miRNAs represent a minority of the non-coding transcriptome, the tangle of lncRNAs is likely to contain as yet unidentified classes of molecules, so lncRNAs as diagnostic tools have properties that are advantageous relative to miRNAs. The above-mentioned characteristics make lncRNAs possible to candidate biomarkers for the diagnosis of CAD. The present study compared the lncRNA profiles between the healthy controls and CAD patients and found that plasma lncRNA *TWF1* was elevated in the patients with CAD and have an 86.2% specificity for CAD.

Recently, the lncRNA-miRNA-mRNA axes were discovered in CVDs including atherosclerosis, myocardial infarction, and cardiomyopathy, and abnormal conditions such as cardiac fibrosis or/and hypertrophy. For example, the nuclear factor IA (*NFIA*) regulates cholesterol homeostasis in the body, promoted progression of atherosclerosis through the lncRNA *RP5833A20.1* sponging miR-382-5p targeting *NFIA* axis [59]. These findings suggest that lncRNAs will be candidates for clinical diagnosis and prognostic markers, providing new therapeutic targets for CVDs and providing insights for the prevention and treatment of other diseases in the future. In the current study, we found that several DEGs were associated with CVDs, just as *C9orf72* and *MBL2* [60, 61], and as the target for hsa-miR-142-3p and hsa-miR-126-5p. Previous research showed that circulating miR126 levels correlated with fasting blood glucose and hemoglobin A1c may lead to CVD [62], and hsa-miR-142-3p up-regulation of miR-142-3p enhances endothelial cell apoptosis and atherosclerotic development which may also contribute to CVD [63]. Two of these miRNAs can be regulatory by *TWF1*. This result indicates that *TWF1* may be a good predictive candidate biomarker for the diagnosis of CAD.

We have to admit the limitation of this study. First, the patients in this study were from one hospital, it is not known whether there is a difference for patients from different areas and races. Therefore, its validity should be tested further in more prospective cohorts. Second, the specific mechanism of lncRNAs-miRNAs-mRNAs axes for regulating the pathogenesis of CAD has not been validated in vivo and in vitro.

## Conclusion

Based on the ceRNA hypothesis, construction of lncRNA-miRNA-mRNA triple regulatory network was used to study molecular mechanism of CAD by to accomplish this, we downloaded 9 datasets from GEO and constructed network by combining differentially expressed lncRNAs, miRNAs and mRNAs. After function analysis and predictive ncRNA network construction, we identified regulatory network contained 15 mRNAs, 3 miRNAs, and 1 lncRNA and 19 edges. To further determine the major regulators in this subnetwork, the topological features, function validation and diagnostic prediction were performed which identified one key lncRNA, *TWF1*.

## Additional file

Additional file 1: **Table S1**. PCR primers for quantitative real-time PCR. **Table S2**. Differentially expressed mRNAs (DEGs). **Table S3**. Differentially expressed miRNAs (DEMis). **Table S4**. Differentially expressed lncRNAs (DELs). (XLSX 113 kb)

## Data Availability

The datasets used and/or analysed during the current study are available from the corresponding author on reasonable request.

## Abbreviations

Apo: Apolipoprotein
BMI: Body mass index
CAD: Coronary artery disease
DBP: Diastolic blood pressure
DEGs: Differentially expressed mRNAs
DELs: Differentially expressed lncRNAs
DEMis: Differentially expressed miRNAs
DO: Disease ontology
HDL-C: High-density lipoprotein cholesterol
LDL-C: Low-density lipoprotein cholesterol
MCODE: Molecular complex detection
PP: Pulse pressure
RMA: Robust Multi-array Average
ROC: Receiver operating characteristic
SBP: Systolic blood pressure
TC: Total cholesterol
TG: Triglyceride
WC: waist circumference
